# Age at onset of type 1 diabetes between puberty and 30 years old is associated with increased diabetic nephropathy risk

**DOI:** 10.1038/s41598-024-54137-2

**Published:** 2024-02-13

**Authors:** Yen-Bo Lin, Taiwan Diabetes Registry Study Group, Wayne Huey-Herng Sheu, Su-Huey Lo, Yen-Po Yeh, Chien-Ning Huang, Chii-Min Hwu, Chang-Hsun Hsieh, Horng-Yi Ou, Lee-Ming Chuang, Jung-Fu Chen, Yu-Cheng Chen, Yun-Hsing Peng, Szu-Tah Chen, Shang-Ren Hsu, Yi-Ling Hsieh, Chih-Hsun Chu, Chieg-Hsiang Lu, Yau-Jiunn Lee, Hua-Fen Chen, Ching-Chu Chen, Chun-Chuan Lee, Pi-Jung Hsiao, Shih-Tzer Tsai, Samuel Chen, Ching-Chieh Su, Yu-Ling Lin, Cho-Tsan Bau, Chung-Chia Liao, Tsung-yung Kuo, Huey-Jen Chen, Chih-Chien Wang, Chiu-Jung Cheng, Yung-Lung Lin, Shin-Chueh Chen, Chung-Yuan Chen, Hsin-Yang Huang, Jiunn-Rong Chen, Hsiao-Jung Lo, Neng-Chun Yu, Wen-Cheng Liu, Chun-Han Wu, Deng-Wang Chen, De-Chung Shen, Wei-Chen Chung, Tien-Jyun Chang

**Affiliations:** 1 Division of Endocrinology and Metabolism, Department of Internal Medicine, National Taiwan University Hospital Bei-Hu Branch, Taipei, Taiwan; 2 Division of Endocrinology and Metabolism, Department of Internal Medicine, National Taiwan University Hospital, Taipei, Taiwan; 3 National Taiwan University School of Medicine, Taipei, Taiwan; 4 Division of Endocrinology and Metabolism, Department of Internal Medicine, Taichung Veterans General Hospital, Taichung, Taiwan; 5 Department of Internal Medicine, School of Medicine, National Yang Ming Chiao Tung University, Taipei, Taiwan; 6 Section of Endocrinology and Metabolism, Department of Internal Medicine, Taipei Veterans General Hospital, Taipei, Taiwan; 7 Institute of Medical Technology, College of Life Science, National Chung-Hsing University, Taichung, Taiwan; 8 National Health Research Institutes, Zhunan Township, Miaoli County Taiwan; 9 Tao-Yuan General Hospital, Ministry of Health and Welfare, Taoyuan City, Taiwan; 10 Changhua County Public Health Bureau, Changhua, Taiwan; 11 Institute of Medicine, Chung Shang Medical University, Taichung, Taiwan; 12 Division of Endocrinology and Metabolism, Department of Internal Medicine, Chung Shang Medical University Hospital, Taichung, Taiwan; 13 Division of Endocrinology and Metabolism, Department of Internal Medicine, Tri-Service General Hospital, Taipei, Taiwan; 14 Department of Internal Medicine, School of Medicine, College of Medicine, National Cheng Kung University, Tainan City, Taiwan; 15 Division of Endocrinology and Metabolism, Department of Internal Medicine, National Cheng Kung University Hospital, Tainan City, Taiwan; 16 Graduate Institute of Clinical Medicine, National Taiwan University College of Medicine, Taipei, Taiwan; 17 Kaoshiung Chang Gung Memorial Hospital, Kaoshiung, Taiwan; 18 Yuanlin Christian Hospital, Changhua, Taiwan; 19 Chiayi Chang Gung Memorial Hospital, Chiayi, Taiwan; 20 Linkou Chang Gung Memorial Hospital, Taoyuan, Taiwan; 21 Changhua Christian Hospital, Changhua, Taiwan; 22 SinLau Hospital, Tainan, Taiwan; 23 Kaohsiung Veterans General Hospital, Kaohsiung, Taiwan; 24 Ditmanson Medical Foundation Chia-Yi Christian Hospital, Chiayi, Taiwan; 25 Lee Yau-Jiunn Clinic, Pingtung, Taiwan; 26 Far Eastern Memorial Hospital, Taipei, Taiwan; 27 China Medical University Hospital, Taichung, Taiwan; 28 MacKay Memorial Hospital, New Taipei City, Taiwan; 29 Kaohsiung Medical University Chung-Ho Memorial Hospital, Kaohsiung, Taiwan; 30 Cheng Hsin General Hospital, Taipei, Taiwan; 31 Samuel Chen Clinic, Nantou, Taiwan; 32 Dr. Su Diabetes Clinic, New Taipei City, Taiwan; 33 Feng-Yuan Hospital, Ministry of Health and Welfare, Taichung, Taiwan; 34 Taichung Hospital, Ministry of Health and Welfare, Taichung, Taiwan; 35 Liao Chung Chia Clinic, Taichung, Taiwan; 36 Ansn Clinic, Hsinchu City, Taiwan; 37 Landseed Hospital, Taoyuan, Taiwan; 38 Show Chwan Memorial Hospital, Changhua, Taiwan; 39 Geng Sin Clinic, Changhua, Taiwan; 40 Shigang District Public Health Center, Taichung, Taiwan; 41 Cheng Ching Hospital, Taichung, Taiwan; 42 Jing Pin Clinic, Kaohsiung, Taiwan; 43 Huang Hsin Yang Clinic, Taitung, Taiwan; 44 Changhua Christian Hospital Yuan Branch, Yunlin, Taiwan; 45 Wei-Gong Memorial Hospital, Miaoli, Taiwan; 46 Neng-Chun Diabetes Clinic, Yilan, Taiwan; 47 Neihu Cathay Clinic, Taipei, Taiwan; 48 Da Chia Clinic, Kaohsiung, Taiwan; 49 Yuan Cheng Clinic, Kaohsiung, Taiwan; 50 Taipei Shen’s Diabetes Center, Taipei, Taiwan; 51 Tseng Han-Chi’s General Hospital, Nantou, Taiwan

**Keywords:** Risk factors, Diabetic nephropathy, Type 1 diabetes, Onset age of type 1 diabetes, Puberty, Diabetes, Diabetes complications, Type 1 diabetes, Chronic kidney disease, Risk factors

## Abstract

Diabetic nephropathy is a critical complication of patients with type 1 diabetes, while epidemiological studies were scarce among Asian countries. We conducted a cross-sectional study to identify factors associated with diabetic nephropathy by questionnaires, using student’s t-test, chi-square test, and multivariable logistic regression. Among 898 participants, 16.7% had diabetic nephropathy. Compared with non-diabetic nephropathy patients, the patients with diabetic nephropathy had significantly higher percentage with onset age of type 1 diabetes between puberty and under 30 years old (female ≥ 12 or male ≥ 13 years old to 29 years old), longer diabetes duration, having family history of diabetes and diabetic nephropathy, accompanied with hypertension, hyperlipidemia, or coronary artery disease (CAD). Compared with patients with onset age before puberty, the odds of diabetic nephropathy occurrence increased to 1.61 times in patients with onset age between puberty and under 30 years old (p = 0.012) after adjusting diabetes duration. Age of diabetes onset between puberty and under 30 years old, diabetes duration, HbA1c, hospital admission within 3 years, diabetic retinopathy, hypertension, systolic blood pressure (SBP), triglyceride levels, and use of angiotensin converting enzyme inhibitor (ACEI) and/or angiotensin receptor blockers (ARB) were independent factors associated with diabetic nephropathy Screening for proteinuria is important in daily clinical practice and should be part of diabetes self-management education for patients with type 1 diabetes.

## Introduction

Type 1 diabetes is one of the most common autoimmune diseases among children, with a global incidence rate increasing to 3% annually since 1990^1,2^. Patients with type 1 diabetes suffer from early destruction of insulin-producing beta-cells of pancreas, which leads to hyperglycemia and several other metabolism-related complications such as cardiovascular disease, retinopathy, nephropathy, and peripheral neuropathy^3^. These complications usually correlate with the duration of diabetes and glycemic control status, and those are closely associated with a higher mortality rate^4^. According to the Taiwan’s National Health Insurance (NHI) claims database (1999–2010), the standardized mortality ratio in patients with type 1 diabetes is approximately three times compared with that of the general population of the same age^5^. Since patients with type 1 diabetes are usually diagnosed at a younger age, the economic burden and healthcare costs could be enormous.

Among complications associated with type 1 diabetes, diabetic nephropathy is the most prevalent. In type 1 and type 2 diabetes cohorts of Swedish and Norwegian populations, the prevalence of cardiovascular disease was similar in type 1 and type 2 diabetes across age groups, whereas diabetic nephropathy was more common in type 1 than type 2 diabetes patients^6^. According to earlier studies, approximately 20–30% of type 1 diabetes patients developed microalbuminuria after a mean diabetes duration of 15 years, and approximately 25–40% of patients with type 1 diabetes ultimately developed diabetic nephropathy^7^. Based on Taiwan’s National Health Insurance (NHI) claims database (1999–2012), the cumulative incidence of nephropathy 12 years after type 1 diabetes diagnosis was 30.2%^8^. In another Taiwan NHI claims database (2000–2016), the prevalence of diabetic nephropathy in patients with type 1 diabetes increased from 10.78 to 20.44% in male and from 12.24 to 22.14% in female patients^9^. The proportion of patients undergoing dialysis and renal transplantation was also high in patients with type 1 diabetes^9^. According to the Diabetes Control and Complications Trial (DCCT) cohort, incident reduced eGFR (eGFR < 60 mL/min/1.73 m^2^), increased mean HbA1c, increased mean triglyceride level, older age, and higher systolic blood pressure (SBP) were the most significant risk factors of diabetic nephropathy^10^.

Age at onset of diabetes has been reported to play an important role in classifying different phenotypes of type 1 DM, namely classical childhood-onset type, or latent adult-onset type (latent autoimmune diabetes in adults, LADA)^11^. A nationwide report in United States also demonstrated two peaks of incident type 1 DM, including during their puberty (aged 11–14 years) and early adulthood^12^. However, these two periods may not share the same pathogenesis in triggering diabetic nephropathy. Physiologically, the surge in growth hormone and sex steroids during puberty contributed to insulin resistance, which therefore accelerate type 1 DM onset^13,14^. One study found that patients with type 1 diabetes with onset age during puberty (age 10–14 years) would have higher risk of developing diabetic nephropathy than those with onset age during pre-puberty (aged 0–9 years)^15^. On the other hand, the renal outcome among LADA seemed to be controversial. One nationwide type 1 diabetes cohort revealed the prevalence of end stage kidney disease were higher in older onset group (> 40 years) than childhood-onset (< 20 years) one^16^. However, another recent cohort indicated type 1 DM diagnosed at childhood/adolescent (age < 20 years) was independently associated with diabetic nephropathy, compared with older onset group (age 30 to 40 years^17^. Since patients diagnosed with LADA not necessarily required insulin injection in the initial period, it should be considered as a mixture of type 1 and type 2 diabetes^18^.

Prevention and early detection of diabetic nephropathy is substantial to patients with type 1 diabetes. However, the incidence of type 1 DM in Western Pacific is relatively low, and studies on nephropathy in patients with type 1 diabetes in Asia are scarce. According to International Diabetes Federation (IDF) Atlas report (10th edition)^19^, the incidence of age standardized type 1 diabetes in Taiwan is less than 10.0 per 100,000 population per year, which is almost one-third of United states or most European countries. To study the prevalence and possible associated risk factors of diabetic nephropathy in patients with type 1 diabetes in Taiwan, we conducted a cross-sectional study by obtaining questionnaires from patients with type 1 diabetes who were registered in the Taiwan Diabetes Registry Study of Diabetes Association of the Republic of China (DAROC, Taiwan) from October 2015 to December 2018.

## Methods

### Participants of Taiwan diabetes registry study

**Figure 1:**
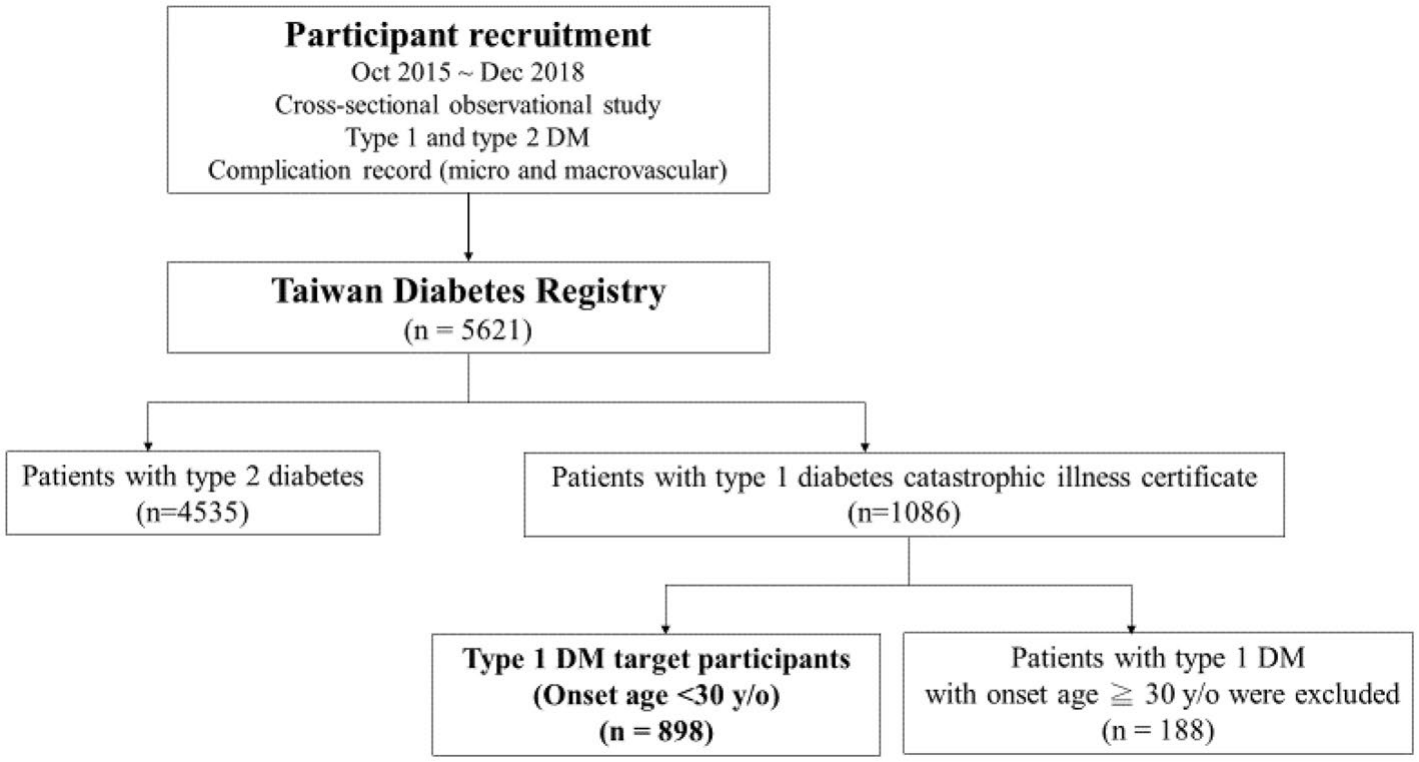
Flowchart of recruitment of patients with type 1 diabetes (onset age < 30 y/o) in Taiwan Diabetes Registration.

The Taiwan Diabetes Registry is a cross-sectional observational study conducted by the Diabetes Association of the Republic of China (Taiwan) to assess the status of healthcare, risk factors, and diabetes-related complications in patients with type 1 and type 2 diabetes. A total of 45 sites (hospitals or primary care clinics) participated in this study and the enrollment of study subjects began in October 2015. The study protocol was approved by the Joint Institutional Review Board and the Institutional Review Board of each hospital (Taichung Veterans General Hospital and National Taiwan University Hospital as representative) participating in this study, (Full detail listed in the Appendix) This study was conducted in accordance with the Declaration of Helsinki and all the participants provided written informed consent in each study subject. In this cross-sectional study, we obtained questionnaires from 1086 patients with type 1 diabetes who were registered in the Taiwan Diabetes Registry Study between October 2015 and December 2018. Because LADA may be the mixture of both type 1 and type 2 diabetes, we excluded those with age of onset over or equal to 30 years old, and 898 participants were recruited finally (Fig. 1). In addition, based on the previous reviews, we divided our groups into pre-puberty and puberty to under 30 group by age at onset of type 1 diabetes. One recent study in Taiwan defined puberty stage as 12 years old in male and 11 years old in female^20^. Because the generation of our cohort is different from the above-mentioned study cohort, after adjustment of age shifting from the above-mentioned cohort (i.e., our cohort is about ten years older than the above-mentioned cohort), we set our puberty group as female ≥ 12 years old and male ≥ 13 years old. Type 1 diabetes was diagnosed in these patients using the Taiwan National Health Insurance Research Database (NHIRD), which has a coverage rate of over 99.9% of the population in Taiwan^21^. Patients diagnosed with type 1 diabetes receiving a certificate for catastrophic illness were recruited in this study. In this questionnaire, each participant’s anthropometric, socioeconomic, and biochemical characteristics were collected by the medical staff. Each patient was requested to provide a urine dipstick test to investigate protein (for overt proteinuria) and/or urine albumin-creatinine ratio (UACR, for microalbuminuria) for diagnosis of diabetic nephropathy. The definition of diabetic nephropathy for this study was based on the clinical guidelines for diabetic management in chronic kidney disease (KDIGO 2020)^22^. According to these guidelines, diabetic nephropathy is mainly determined by estimated glomerular filtration rate (eGFR) < 60 (ml/min/1.73 m) or persistent albuminuria (UACR ≥ 30 mg/g)^23^. For patients without data on the status of albuminuria, we used protein findings from the urine dipstick test, wherein trace and greater proteinuria (trace to +, ++, +++, and ++++) would fall into albuminuria categories A2 and A3, respectively (A2: microalbuminuria, 30–300 mg/g, dipstick trace to ++, A3: macroalbuminuria, > 300 mg/g, dipstick: +++ to ++++)^23^. According to this urine dipstick conversion model, the sensitivity and specificity for detecting a UACR of ≥ 30 mg/g was 62% and 87.7%, respectively ^23^.

### Statistical analyses

The student’s t-test and chi-square test were used to compare the differences in various continuous and categorical variables between patients with and without diabetic nephropathy, respectively. Multivariate logistic regression was used to identify the diabetic nephropathy associated factors. A two-sided p < 0.05 level was considered statistically significant. All analyses were performed using Stata Statistical Software: Release 14 (StataCorp. 2015. College Station, TX: StataCorp LP).

### Conference presentation

Parts of this study were presented as oral presentation on March 28, 2021 at the 42nd Annual Meeting of the Endocrine Society and the Diabetes Association of the R.O.C (Taiwan) held in Taipei, Taiwan. Parts of this study were also presented as e-poster at the International Diabetes Federation (IDF) Virtual Congress 2021 held between 6 and 11th, December, 2021.

## Results

### Anthropometric, and biochemical characteristics in non-diabetic nephropathy and diabetic nephropathy patients with type 1 diabetes

Among the 898 participants, 150 (16.7%) were diagnosed with diabetic nephropathy. The median age was 28 years and 59.9% of the participants were female. The mean age of the diabetic nephropathy group was statistically greater than that of the non-diabetic nephropathy group (33.0 ± 10.5 vs. 28.1 ± 11.1 years, p = 0.001) (Table 1). (this paragraph has moved to method section). Among type 1 diabetes patients with diabetic nephropathy, the proportion of puberty to the under-30-year-old onset group was statistically higher than that of participants without diabetic nephropathy (56.9% in diabetic nephropathy participants vs. 45.5% in non-diabetic nephropathy participants, p = 0.028). In the diabetic nephropathy group, 57.3%, 18.7%, 6.7%, 8%, 4%, and 5.3% of patients were classified as Grade 1, 2, 3a, 3b, 4, and 5 KDIGO GFR category, respectively. The mean duration after type 1 diabetes diagnosed in the diabetic nephropathy group was statistically longer than that in the non-diabetic nephropathy group (17.6 ± 9.9 vs. 13.9 ± 9.3 years, p < 0.001). The duration of type 1 diabetes with diabetic nephropathy was significantly longer at both pre-puberty onset group and puberty to under 30-year-old onset group (pre-puberty: 20.3 ± 9.4 years vs. 14.9 ± 8.6 years, p < 0.001; puberty to under 30 years old: 16.2 ± 10.0 years, 13.3 ± 9.7 years, p = 0.009). With respect to diabetic nephropathy associated risk factors, patients with diabetic nephropathy had a higher prevalence of hypertension (21.3% vs. 5.2%, p < 0.001), hyperlipidemia (16.7% vs. 5.6%, p < 0.001), positive family history of diabetes (48% vs. 32.6%, p < 0.001), and a positive family history of CKD (11.3% vs. 5.9%, p = 0.028). Notably, 23.1% of the non-diabetic nephropathy participants and 11.3% of the diabetic nephropathy participants did not provide data on family history of CKD. Compared with patients without diabetic nephropathy, those with diabetic nephropathy were more likely to have coronary artery disease (CAD) (3.4% vs. 0.9%, p = 0.038) and heart failure (1.3% vs. 0%, p = 0.028). Similarly, the diabetic nephropathy group had a higher percentage of patients with diabetic retinopathy requiring laser therapy (30% vs. 2.9%, p < 0.001), maculopathy (3.3% vs. 0.5%, p = 0.009), non-proliferative retinopathy (12.7% vs. 7.1%, p = 0.022), and proliferative retinopathy (14.7% vs. 1.7%, p < 0.001). With respect to hospital admission, patients with diabetic nephropathy were more likely to be hospitalized in the past 3 years (46.7% vs. 25.7%, p = 0.035). On the other hand, compared with participants in the non-diabetic nephropathy group, a higher percentage of participants with diabetic nephropathy received antihypertensive drugs, including ACEI (6.7% vs. 0.9%, p < 0.001) and ARB (18.7% vs. 4.0%, p < 0.001). With respect to anthropometric characteristics, diabetic nephropathy patients had a significantly greater waist circumference (80.1 ± 11.8 vs. 76.4 ± 10.2 cm, p < 0.001), higher systolic blood pressure (SBP) and diastolic blood pressure (DBP) (SBP, 126.2 ± 19.1 vs. 117.9 ± 14.8 mmHg, p < 0.001; DBP, 76.4 ± 13.3 vs. 70.9 ± 9.8 mmHg, p < 0.001) compared with non-diabetic nephropathy patients. Regarding biochemical characteristics, diabetic nephropathy patients had higher HbA1c levels (8.7 ± 2.1% vs. 8.1 ± 1.6%, p < 0.001), higher serum creatinine (124.1 ± 179.5 vs. 62.9 ± 14.4 µmol/L, p < 0.001), lower eGFR (95.7 ± 45.7 vs. 119.3 ± 30.1 ml/min/1.73 m^2^, p < 0.001), higher triglyceride (3.1 ± 6.9 vs. 1.9 ± 1.5 mmol/l, p < 0.001), and higher total cholesterol (4.9 ± 1.3 vs. 4.6 ± 0.8 mmol/l, p = 0.002) compared with non-diabetic nephropathy participants (Table 1).

**Table 1:** Anthropometric, socioeconomic, and biochemical characteristics of diabetic nephropathy and non-diabetic nephropathy group in type 1 DM patients in Taiwan Diabetes Registry.

	Non-diabetic nephropathy (n = 748)	Diabetic nephropathy (n = 150)	p-value
Epidemiologic characteristics			
Age, year	28.1 ± 11.1	33.0 ± 10.5	0.001
Onset age of type 1 diabetes, year			0.028
Pre-puberty (female < 12/male < 13)	332 (36.3%)	52 (30.2%)	
Puberty (female:12/male:13) to under 30	416 (45.5%)	98 (56.9%)	
Male sex, n (%)	309 (41.3%)	61 (40.7%)	0.884
GFR category, n (%)			0.000
Grade 1	665 (88.9%)	86 (57.3%)	
Grade 2	83 (11.1%)	28 (18.7%)	
Grade 3a	0	10 (6.7%)	
Grade 3b	0	12 (8%)	
Grade 4	0	6 (4%)	
Grade 5	0	8 (5.3%)	
Type 1 DM duration, year	13.9 ± 9.3	17.6 ± 9.9	< 0.001
Pre-puberty (female < 12/male < 13)	14.9 ± 8.6	20.3 ± 9.4	< 0.001
Puberty (female:12/male:13) to under 30	13.3 ± 9.7	16.2 ± 10.0	0.009
CKD associated history			
Diabetic risk factors, n (%)			
Obesity	44 (5.9%)	10 (6.7%)	0.136
F.H. of diabetes	244 (32.6%)	72 (48%)	< 0.001
F.H. of CKD	34 (5.9%)	15 (11.3%)	0.028
Didn’t know or uncertain	17 (2.3%)	3 (2%)	0.564
Diabetes comorbidity, n (%)			
Hypertension	39 (5.2%)	32 (21.3%)	< 0.001
Hyperlipidemia	42 (5.6%)	25 (16.7%)	< 0.001
Diabetes macrovascular and microvascular complications			
Cardiovascular disease, n (%)			
CAD	7 (0.9%)	5 (3.4%)	0.038
HF	0 (0%)	2 (1.3%)	0.028
Arrythmia	5 (0.7%)	1 (0.6%)	0.736
Uncertain	2 (0.3%)	0 (0%)	0.694
Cerebrovascular disease, n (%)			
Ischemic stroke	0 (0%)	0 (0%)	0.833
Hemorrhagic disease	1 (0.1%)	0 (0%)	
Didn’t know	0 (0%)	0 (0%)	
Peripheral vascular disease, n (%)	0 (0%)	0 (0%)	
Retinopathy, n (%)			
Maculopathy	4 (0.5%)	5 (3.3%)	0.009
Non-proliferative retinopathy	53 (7.1%)	19 (12.7%)	0.022
Proliferative retinopathy	13 (1.7%)	22 (14.7%)	< 0.001
Blindness	2 (0.3%)	1 (0.7%)	0.422
Laser therapy for diabetes retinopathy	22 (2.9%)	45 (30%)	< 0.001
Hospitalization in past 3 years, n (%)			0.035
None	537 (71.8%)	78 (52%)	
Yes	192 (25.7%)	70 (46.7%)	
Didn’t know or uncertain	19 (2.5%)	2 (1.3%)	
Anti-hypertensive drugs			
ACEI	7 (0.9%)	10 (6.7%)	< 0.001
ARB	30 (4%)	28 (18.7%)	< 0.001
Anthropometric characteristics			
Physical examination			
Body height (cm)	162.6 ± 9.8	162.9 ± 7.7	0.692
Body weight (kg)	60.6 ± 12.5	61.6 ± 13.1	0.365
BMI (kg/m^2^)	22.8 ± 3.9	23.1 ± 4.2	0.334
SBP (mmHg)	117.9 ± 14.8	126.2 ± 19.1	< 0.001
DBP (mmHg)	70.9 ± 9.8	76.4 ± 13.3	< 0.001
Waist circumference (cm)	76.4 ± 10.2	80.1 ± 11.8	< 0.001
Hip circumference (cm)	93 ± 9	94.2 ± 8.9	0.155
Waist hip ratio (WHR)	0.65 ± 0.09	0.65 ± 0.09	0.764
Biochemical characteristics			
Fasting plasma glucose (mmol/L)	9.5 ± 4.2	9.4 ± 4.5	0.946
HbA1c (%)	8.1 ± 1.6	8.7 ± 2.1	< 0.001
Creatinine (µmol/L)	62.9 ± 14.4	124.1 ± 179.5	< 0.001
eGFR (ml/min)	119.3 ± 30.1	95.7 ± 45.7	< 0.001
Triglyceride (mmol/L)	1.9 ± 1.5	3.1 ± 6.9	< 0.001
Total cholesterol (mmol/L)	4.6 ± 0.8	4.9 ± 1.3	0.002
LDL (mmol/L)	2.5 ± 0.7	2.6 ± 0.9	0.200
ALT (U/l)	18.3 ± 18.3	20.1 ± 15.5	0.261

*GFR* Glomerular filtration rate, *DM* diabetes mellitus, *FH* Family history, *DM* diabetes mellitus, *CKD* Chronic kidney disease, *CVD* cardiovascular disease, *DKA* diabetic ketoacidosis, *CAD* coronary artery disease, *HF* heart failure, *ACEI* Angiotensin Converting Enzyme inhibitors, *ARB* angiotensin receptor blockers, *BMI* Body mass index, *SBP* systolic blood pressure, *DBP* Diastolic blood pressure, *HbA1c* Hemoglobulin A1c, *eGFR* estimated Glomerular filtration rate, *LDL* Low-density lipoprotein, *ALT* alanine transaminase.

### Associated factors for diabetic nephropathy in patients with type 1 diabetes

To further investigate specific factors associated with diabetic nephropathy in patients with type 1 diabetes, univariate logistic regression was individually applied to the identified statistically significant variables in Table 1. Older age (OR 95% CI 1.04, 1.02–1.05; p < 0.001) and a longer duration of type 1 DM (OR 95% CI 1.04, 1.02–1.06; p < 0.001) were positively associated with the presence of diabetic nephropathy. Compared with pre-puberty onset group, patients diagnosed as type 1 diabetes between puberty (female ≥ 12 years/male ≥ 13 years) and under 30 years old are more likely to have diabetic nephropathy (OR 95% CI 1.61, 1.11–2.34, p = 0.012) after adjusting diabetes duration (Table 2). In addition, type 1 diabetes patients with a family history of CKD (OR 95% CI 2.02, 1.07–3.83; p = 0.031) and diabetes (OR 95% CI 1.91, 1.34–2.72; p < 0.001) had higher odds of the presence of diabetic nephropathy. Patients who had hypertension (OR 95% CI 4.93, 2.97–8.18; p < 0.001), hyperlipidemia (OR 95% CI 3.36, 1.98–5.71; p < 0.001), CAD history (OR 95% CI 3.59, 1.12–11.5; p = 0.031), higher SBP (OR 95% CI 1.03, 1.02–1.04; p < 0.001), DBP (OR 95% CI 1.05, 1.03–1.07; p < 0.001), HbA1c levels (OR 95% CI 1.22, 1.11–1.35; p < 0.001), triglyceride (OR 95% CI 1.19, 1.08–1.30; p < 0.001), total cholesterol (OR 95% CI 1.30, 1.09–1.57; p = 0.003) and waist circumference (OR 95% CI 1.03, 1.02–1.05; p < 0.001) were all positively associated with the presence of diabetic nephropathy. Presence of retinopathy was also positively associated with the presence of diabetic nephropathy in patients with type 1 diabetes in a severity-dependent manner (compared with no diabetic retinopathy, non-proliferative retinopathy: OR 95% CI 2.41, 1.37–4.23; p = 0.002 and for proliferative retinopathy: OR 95% CI 12.1, 5.79–25.1; p < 0.001). Hospitalization in the past 3 years was positively associated with the presence of diabetic nephropathy in patients with type 1 diabetes (OR 95% CI 1.86, 1.37–2.54; p < 0.001). Furthermore, patients taking ACEI (OR 95% CI 7.56, 2.83–20.2; p < 0.001), ARB (OR 95% CI 5.49, 3.17–9.52; p < 0.001), and either of these (OR 95% CI 6.52, 3.98–10.7; p < 0.001) had higher odds for the presence of diabetic nephropathy (Table 2).

**Table 2:** Association between specific factors and the presence of diabetic nephropathy in patients with type 1 diabetes.

Variable	Odds ratio (95% CI)	p value
Age	1.04 (1.02–1.05)	< 0.001
Age of onset		
Pre-puberty (female < 12/male < 13)	1 (Ref)	
Puberty (female:12/male:13) to under 30	1.61 (1.11–2.34)	0.012
Type 1 DM duration	1.04 (1.02–1.06)	< 0.001
Diabetes risk factors		
F.H. of DM	1.91 (1.34–2.72)	< 0.001
F.H. of CKD	2.02 (1.07–3.83)	0.031
Diabetes comorbidity		
Hypertension	4.93 (2.97–8.18)	< 0.001
Hyperlipidemia	3.36 (1.98–5.71)	< 0.001
DM complications		
CAD	3.59 (1.12–11.5)	0.031
Retinopathy		
None	1.00 (Reference)	
Non-proliferative retinopathy	2.41 (1.37–4.23)	0.002
Proliferative retinopathy	12.1 (5.79–25.1)	< 0.001
Hospitalization in past 3 years	1.86 (1.37–2.54)	< 0.001
Antihypertensive drugs		
ACEI	7.56 (2.83–20.2)	< 0.001
ARB	5.49 (3.17–9.52)	< 0.001
ACEI or ARB	6.52 (3.98–10.7)	< 0.001
SBP	1.03 (1.02–1.04)	< 0.001
DBP	1.05 (1.03–1.07)	< 0.001
Waist circumference	1.03 (1.02–1.05)	< 0.001
HbA1c	1.22 (1.11–1.35)	< 0.001
Total cholesterol	1.30 (1.09–1.57)	0.003
Triglyceride	1.19 (1.08–1.30)	< 0.001

*DM* diabetes mellitus, *FH* Family history, *DM* diabetes mellitus, *CKD* chronic kidney disease, *CAD* coronary artery disease, *ACEI* Angiotensin Converting Enzyme inhibitors, *ARB* angiotensin receptor blockers, *SBP* systolic blood pressure, *DBP* Diastolic blood pressure, *HbA1c* Hemoglobulin A1c.

### Independent factors associated with the presence of diabetic nephropathy in patients with type 1 diabetes

A multivariate stepwise logistic regression model was applied to the statistically significant variables found in Table 2 to identify independent factors associated with the presence of diabetic nephropathy in patients with type 1 diabetes (Table 3). Those who diagnosed type 1 DM between puberty (female ≥ 12/male ≥ 13) and under 30 years old (OR 95% CI 1.56, 1.05–2.34; p = 0.029) were positively related to the presence of diabetic nephropathy compared with those with pre-puberty onset. Longer diabetes duration (OR 95% CI 1.03, 1.01–1.05; p = 0.005), higher HbA1c levels (OR 95% CI 1.23, 1.09–1.37; p < 0.001), hospitalization in past 3 years (OR 95% CI 1.95, 1.39–2.74; p < 0.001), diabetic retinopathy (OR 95% CI 3.12, 1.95–4.99; p < 0.001), higher SBP (OR 95% CI 1.03, 1.02–1.04; p < 0.001), higher triglyceride (OR 95% CI 1.12, 1.01–1.24; p = 0.037) were associated with a higher risk of the presence of diabetic nephropathy (Table 3).

**Table 3:** Independent factors associated with the presence of diabetic nephropathy in patients with type 1 diabetes using stepwise multivariate logistic regression.

Variable	Odds ratio (95% CI)	p value
Age of onset (female:12/male:13 to under 30)	1.56 (1.05–2.34)	0.029
Duration	1.03 (1.01–1.05)	0.005
HbA1c	1.23 (1.09–1.37)	< 0.001
Hospitalization in past 3 years	1.95 (1.39–2.74)	< 0.001
Diabetic retinopathy	3.12 (1.95–4.99)	< 0.001
SBP (mmHg)	1.03 (1.02–1.04)	< 0.001
Triglyceride (mmol/l)	1.12 (1.01–1.24)	0.037

*HbA1c* Hemoglobulin A1c, *SBP* systolic blood pressure, *ACEI* Angiotensin Converting Enzyme inhibitors, *ARB* angiotensin receptor blockers.

## Discussion

In this registry study, the prevalence of diabetic nephropathy in patients with type 1 diabetes in Taiwan was 16.7%, with a mean diabetes duration of 14.5 years. Longer duration, higher HbA1c, SBP, and plasma triglyceride level, diabetic retinopathy, hospitalization in the past 3 years were independently associated with the presence of diabetic nephropathy in patients with type 1 diabetes. Age at onset of type 1 diabetes between puberty and under 30 years old was also independently associated with higher presence rate of diabetic nephropathy after adjusting diabetes duration. To the best of our knowledge, this is the first Asian-Pacific multicenter diabetes registration study that demonstrated type 1 diabetes diagnosed between puberty and under 30 years old is associated with higher prevalence of diabetic nephropathy.

Compared with our cohort, one prospective study from the UK indicated that the cumulative incidence of microalbuminuria was 25.7% and 50.7% after 10 and 19 years of diabetes, respectively^24^. This difference in incidence may be attributed to both genetic susceptibility and environmental triggers^25,26^. In the recent Genome-Wide Association Study^27^, Caucasian and Chinese populations have been shown to share different type 1 diabetes risk loci, and several environmental factors were identified to affect the incidence of type 1 DM^28^. Another important factor affected diabetic nephropathy risk was the onset age of type 1 diabetes. Patients with type 1 diabetes diagnosed between puberty and under 30 years old had higher odds compared with pre-puberty onset group (OR 95% CI 1.61, 1.11–2.34; p = 0.012) in this study. In our following stepwise multivariate logistic regression model for diabetic nephropathy, patients who diagnosed type 1 diabetes between puberty and under 30 years old also have higher odds for diabetic nephropathy (OR 95% CI 1.56, 1.05–2.34, p = 0.029). Puberty has been considered as an important but controversial independent risk factor of type 1 diabetic nephropathy. Several studies demonstrated that albuminuria and variation in renal function during puberty changed more rapidly than those in pre-puberty group^29,30^. Although previous studies showed that patients with type 1 diabetes with onset age during puberty would have higher risk of developing diabetic nephropathy than those with onset age during pre-puberty^15,31,32^, these studies only included patients with type 1 diabetes diagnosed during childhood and adolescence, but the patients with type 1 diabetes between 18 and under 30 years old were not investigated. Recent cohort in Finland showed most type 1 diabetes diagnosed younger than 5 years had lower residual serum C-peptide level and progressed faster to absolute insulin deficiency, and was associated with higher HbA1c, higher percentage with nephropathy, retinopathy, and hypertension than older onset groups^33^. However, in Finland diabetes registry, patients with type 1 diabetes diagnosed between 10 and 14 years old group have the highest relative risk of ESRD (compared to age of onset < 5 years old), suggesting pubertal onset of type 1 diabetes may have an accelerating role in kidney damage^34^. In contrast, a Swedish diabetes registry showed that the risk of ESRD seemed different between gender. The age at onset 20–34 years conferred the highest risk of developing diabetic nephropathy in male, but the highest risk of onset age in female was 10–19 years^35^. Another type 1 diabetes cohort in Korea demonstrated patients with type 1 diabetes diagnosed at childhood/adolescent (age < 20 years) had higher prevalence of diabetes nephropathy than in older onset groups, and the degree of eGFR decrease was more prominent in the childhood/adolescent-onset group than in the older onset group^17^. In contrast, a population-based retrospective cohort study of type 1 diabetes in Hong Kong showed that people with type 1 diabetes diagnosed aged older than 40 years had a higher hazards of end stage renal disease (ESRD) versus those diagnosed aged less than 20 years^16^. The above studies on type 1 diabetes cohort showed different onset age group of type 1 diabetes had different prevalence of diabetic nephropathy. Some reported patients diagnosed type 1 diabetes at younger age had higher prevalence of diabetic nephropathy^17,33^, and one showed type 1 diabetes diagnosed older than 40 years had a higher risk to develop ESRD^16^. This discrepancy among the previous studies and our study may be due to the different genetic backgrounds of ethnic difference, the cohort including LADA with the mixture of type 1 and type 2 diabetes, and the grouping of onset age not according to the puberty. It needs further investigation to elucidate the impact of puberty on the development of diabetic nephropathy and decline rate of eGFR in patients with type 1 diabetes. In our study, we found that the onset of type 1 diabetes between puberty and 30 years old was associated with a higher prevalence of diabetic nephropathy, even after adjusting for disease duration, which supported the hypothesis that post-puberty related hormone disturbance would induce insulin resistance, resulting more contribution in diabetic complications than pre-puberty ones. From the treatment point of view, more stringent glycemic and blood pressure control, and early detection of microalbuminuria or deterioration of eGFR are especially important in patients with type 1 diabetes diagnosed between puberty and under 30 years old.

Regarding diabetic nephropathy risk factors, our findings were consistent with Diabetes Control and Complications Trial (DCCT) cohort, which increased mean HbA1c, SBP, increased mean triglyceride level and longer diabetes duration were associated with presenting microalbuminuria^10^. According to Taiwan’s NHI medical claims (1995–2017), in type 1 diabetes patients with a family history of type 2 diabetes, the adjusted hazard ratio for nephropathy was also higher (HR 95% CI 1.44, 1.27–1.64) compared with those without a family history of type 2 DM^36^. Similarly, type 1 DM patients in this study who had a family history of diabetes had higher odds of having diabetic nephropathy (OR 95% CI 1.91, 1.34–2.72; p < 0.001). In this study, a higher percentage of diabetic nephropathy patients had coexisting CAD and heart failure, which may have contributed to a higher percentage of hospitalization in the past 3 years in the diabetic nephropathy group than in the non-diabetic nephropathy group. Diabetic retinopathy, especially in the proliferative retinopathy group, was also more prevalent in type 1 diabetes patients with diabetic nephropathy in our study. According to the literature, the presence of a pre-existing microvascular complication (retinopathy or nephropathy) may contribute to the development of another complication, especially in patients with type 1 diabetes^37^. In the Steno study, which observed 220 patients with type 1 diabetes with and without nephropathy in a 15 year-follow-up study, those developed gross proteinuria were found to have a higher risk of progression to proliferative retinopathy (12% annually) as compared with those without nephropathy (1–2% annually)^38^. In the EURODIAB Complications Study, retinopathy at baseline in type 1 DM patients was positively associated with new-onset microalbuminuria (53.2%) after 15 years, and it was more frequent among proliferative retinopathy group (67.2%)^39^. Chronic hyperglycemia, which is measured by mean blood glucose or HbA1c levels, has been linked to the development and progression of microvascular complications^40^. In this study, we also found that a higher HbA1c level was positively associated with the presence of diabetic nephropathy. In this study, patients with type 1 DM who were taking ACEI or ARB had higher odds for the diagnosis of diabetic nephropathy. Because this registration cohort was a cross-sectional study, only association could be found, but no causal relationship could be determined. This finding may be due to the higher SBP and DBP found in patients with diabetic nephropathy, which led to more patients with diabetic nephropathy receiving ACEI/ARB compared to non-diabetic nephropathy. ACEI and ARB was preferred as first-line therapy to patient with UACR ≥ 30 mg/g to slow down the progression^41^. It is well documented that higher triglyceride and cholesterol levels are associated with a more rapid decline in kidney function^42^. Dyslipidemia may directly affect the kidney by disturbing the renal lipid homeostasis. Further, it indirectly affects the kidneys through systemic inflammation, oxidative stress, and vascular injury^43,44^. A population-based prospective cohort study in Hong Kong showed smoking, increased HbA1c level, hypertension, albuminuria, and dyslipidemia were associated with greater risk of ESRD in patients with youth-onset type 1 diabetes (age at onset of type 1 DM < 20 years old)^45^. The identified risk factors associated with ESRD are similar with the risk factors associated with diabetic nephropathy in patients with type 1 diabetes in our study. However, our study emphasized on the onset age of type 1 diabetes after puberty, instead of only diabetes duration, is associated with higher prevalence of diabetic nephropathy, which was not emphasized in the above study^45^.

Some of the strengths of this study are as follows: first, this was the first nationwide registration cohort of type 1 diabetes which emphasized on the impact of onset age of type 1 diabetes on diabetic nephropathy, especially between puberty to under 30 years old. As we excluded LADA (age of onset ≥ 30 years old) group in our statical analysis, the interference of diabetic nephropathy risk factors (more related to type 2 diabetes) was mitigated. Second, detailed information about anthropometric characteristics, family history, comorbidity, diabetes-related complications, medications, and laboratory data were collected, which greatly decreased the bias of making inappropriate inference.

However, our study also had some limitations. First, since this is a cross-sectional study, the efficacy of causal association may be weaker than that of longitudinal cohort studies. Since we lacked exposure period of drugs and baseline kidney function before recruitment, it is difficult to answer the use of ACEi/ARB was before or after diabetic nephropathy noticed, as well as excluding any systemic factors such as systemic lupus erythematosus which may also lead to proteinuria. However, since type 1 diabetes is a relatively young onset disease, we could only assume that the proteinuria was mostly associated with diabetes. Second, 274 (30.5%) of the total patients lacked urine albumin-to-creatinine ratio (UACR), which may strongly influence our diabetic nephropathy classification. Therefore, we applied a conversion equation from urine dipstick protein to UACR for the missing data, which was validated in a meta-analysis^23^. Third, the mean age of menarche could be estimated with a shift of 0.43 years per decade in previous Taiwan study^20^. However, studies referred to puberty onset shift among male are scarce, which the exact cutoff was difficult to be confirmed. Lastly, the total number of participants in the study was approximately 900, which may not be sufficient for extrapolation of our findings to other type 1 diabetes populations with a higher incidence of type 1 diabetes as well as to populations of other ethnicities. Additional limitation of questionnaire including not specifying type 1 or type 2 diabetes in diabetes family history and hospitalization reason were also noted.

In conclusion, we identified several modifiable risk factors of diabetic nephropathy in patients with type 1 diabetes from the nationwide registration of the type 1 diabetes cohort in Taiwan. These included poor glycemic control, high SBP, and high serum triglyceride levels. In addition, we identified that patients with type 1 diabetes diagnosed between puberty and under 30 years old was associated with higher prevalence of diabetic nephropathy in patients with type 1 diabetes. Finally, we found a lower rate of ACEI or ARB use in type 1 DM patients with diabetic nephropathy, which could be a room for improvement to our daily practice.

Since ACEI or ARB prescription is recommended in patient with diabetic nephropathy in both American Diabetes Association (ADA)^41^ and Diabetes Association of the Republic of China (DAROC) type 1 diabetes guideline^46^, our government has implemented the policy for screening all diabetic patients at least annually for UACR, and strongly suggest further semi-annually follow-up if the result was abnormal. As there were only 20% of prescription rate of ACEI or ARB and short consultation time for doctors spending on patients in Taiwan (less than 5 min)^47^, enhancing the awareness of the clinicians to regular check proteinuria and a pop-up reminder implemented in electronic medical record (EMR) system would be an applicable solution in raising ACEI/ARB prescription rate. All the above findings are informative and will be helpful to emphasize the screening for proteinuria in daily clinical practice and diabetes self-management education to improve the quality of care for patients with type 1 diabetes.

### Supplementary Information

Supplementary Information.

## Data Availability

The datasets generated or analyzed during the current study were available from Diabetes Association of the Republic of China (Taiwan), but restriction was applied to the availability of these data, which indicated that the current datasets were used under license, and therefore not publicly available. However, the datasets could be available from the authors on reasonable requests and with permission of Diabetes Association of the Republic of China (Taiwan).
