# Supplementary material for: Age at onset of type 1 diabetes between puberty and 30 years old is associated with increased diabetic nephropathy risk
Source: Sci Rep. 2024 Feb 13;14:3611. doi: 10.1038/s41598-024-54137-2 (PMC10864267; doi:10.1038/s41598-024-54137-2)
Supplement: Supplementary file 1 — Supplementary Information. [file 41598_2024_54137_MOESM1_ESM.docx]

**Appendix**

Lists of Institutional Review Board of each hospital

Taichung Veterans General Hospital

Tao-Yuan General Hospital

Chung Shang Medical University Hospital

Taipei Veterans General Hospital

Tri-Service General Hospital

National Cheng Kung University Hospital

Kaoshiung Chang Gung Memorial Hospital

Yuanlin Christian Hospital

Chiayi Chang Gung Memorial Hospital

Linkou Chang Gung Memorial Hospital

Changhua Christian Hospital

SinLau Hospital

Kaohsiung Veterans General Hospital.

Ditmanson Medical Foundation Chia-Yi Christian Hospital

National Taiwan University Hospital

Far Eastern Memorial Hospital

China Medical University Hospital

MacKay Memorial Hospital

Kaohsiung Medical University Chung-Ho Memorial Hospital

Cheng Hsin General Hospital

Feng-yuan Hospital, Ministry of Health and Welfare, Taiwan

Taichung Hospital, Ministry of Health and Welfare, Taiwan.

Landseed Hospital

Show Chwan Memorial Hospital

Shigang Distric Public Health Center, Taichung City

Cheng Ching Hospital, Taichung, Taiwan

Changhua Christian Hospital Yuan Branch, Yunlin County, Taiwan

Wei-Gong Memorial Hospital, Miaoli, Taiwan

Tseng Han-Chi's general hospital
